# Enhanced Superconductivity and Critical Current Density Due to the Interaction of InSe_2_ Bonded Layer in (InSe_2_)_0.12_NbSe_2_

**DOI:** 10.1021/jacs.3c09756

**Published:** 2024-01-05

**Authors:** Rui Niu, Jiayang Li, Weili Zhen, Feng Xu, Shirui Weng, Zhilai Yue, Xiangmin Meng, Jing Xia, Ning Hao, Changjin Zhang

**Affiliations:** † High Magnetic Field Laboratory of Anhui Province, HFIPS, Chinese Academy of Sciences, Hefei 230031, China; ‡ Science Island Branch of Graduate School, University of Science and Technology of China, Hefei 230026, China; § Key Laboratory of Photochemical Conversion and Optoelectronic Materials, Technical Institute of Physics and Chemistry, Chinese Academy of Sciences, Beijing 100190, China; ∥ Collaborative Innovation Center of Advanced Microstructures, Nanjing University, Nanjing 210093, China

## Abstract

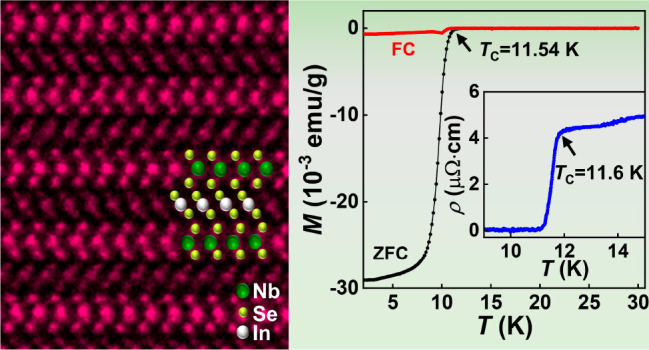

Superconductivity was discovered in (InSe_2_)_*x*_NbSe_2_. The materials are crystallized in a unique layered structure where bonded InSe_2_ layers are intercalated into the van der Waals gaps of 2H-phase NbSe_2_. The (InSe_2_)_0.12_NbSe_2_ superconductor exhibits a superconducting transition at 11.6 K and critical current density of 8.2 × 10^5^ A/cm^2^. Both values are the highest among all transition metal dichalcogenide superconductors at ambient pressure. The present finding provides an ideal material platform for further investigation of superconducting-related phenomena in transition metal dichalcogenides.

The experimental realization of room-temperature superconductors is believed to herald a revolution in electric power technologies, fulfilling a long-standing dream of human beings.^1^ Additionally, the fabrication of superconducting materials with transition temperatures surpassing those of conventional superconductors holds particular importance. Investigating the physical properties of these materials could provide vital clues to establish the pairing mechanisms of superconductivity, ultimately contributing to the realization of room-temperature superconductors.^2,3^ In recent years, significant strides have been made in discovering new superconductors with unique properties. For instance, superconductivity with a transition temperature close to room temperature has been reported in various hydrogen-rich compounds under high pressure.^4^ Unconventional superconductivity has also been observed in magic-angle graphene,^5^ infinite-layer nickel oxides,^6^ and CsV_3_Sb_5_-related compounds.^7−9^

NbSe_2_, a transition metal dichalcogenide (TMD) superconductor, has been a subject of intense investigation over the past several decades.^10−14^ This compound undergoes a charge density wave (CDW) transition at *T*_CDW_ ∼ 40 K and a superconducting transition at *T*_c_ ∼ 7.5 K.^11^ Understanding the interplay between these two phase transitions has become the focus of research.^12−14^ Particularly, it is interesting to investigate whether the superconductivity could be substantially enhanced when the CDW transition is suppressed in this compound.

The intercalation of elements into the van der Waals gaps of layered materials has proven to be an efficient method for achieving superconductivity and regulating the superconducting transition temperature.^15−19^ For instance, by intercalating Cu or Sr into topological insulator Bi_2_Se_3_, the material can be tuned into superconducting Cu(Sr)_*x*_Bi_2_Se_3_.^15,16^ Similarly, the intercalation of Tl and K in FeSe can significantly elevate *T*_c_ from approximately 10 K to around 30 K in (Tl, K)_*x*_Fe_2_Se_2_.^17−19^ In the case of NbSe_2_, various metal elements have been selected for intercalation into its van der Waals gaps. However, in most cases, this process leads to a decrease of *T*_c_.^20−23^ Therefore, it remains a big challenge to fabricate a new superconducting material based on NbSe_2_ that exhibits a superior transition temperature.

Here, we report a remarkable increase in both the superconducting transition temperature and critical current density by the intercalation of indium into NbSe_2_. The resulting materials exhibit superconductivity with a maximum transition temperature of 11.6 K and a critical current density of 8.2 × 10^5^ A/cm^2^, surpassing all other TMD superconductors. Unlike previous studies involving the intercalation of individual atoms into the van der Waals gaps,^15−23^ our atomic-resolution high-angle annular dark-field aberration-corrected scanning transmission electron microscopy (HAADF-STEM) measurements reveal that the intercalated In atoms form InSe_2_ bonds within the van der Waals layers of NbSe_2_, resulting in a unique crystal structure termed (InSe_2_)_*x*_NbSe_2_. The insertion of an InSe_2_ bonded layer provides a new possibility in improving the superconducting performances of TMD-related superconductors.

Figure 1a shows the atomic-resolution HAADF-STEM image of a representative (InSe_2_)_*x*_NbSe_2_ sample within the *ab* plane, which reveals the presence of rhombohedral-arrayed atoms without any stacking defects, indicating the high quality of the single-crystal samples. The determined *a*-axis lattice constant is 3.55 Å, consistent with the in-plane lattice constant of NbSe_2_.^10^ Notably, in the center of some hexagons, we observe additional spots exhibiting relatively less brightness compared to the Nb and Se sites. These spots are attributed to the intercalated In atoms.

**Figure 1:**
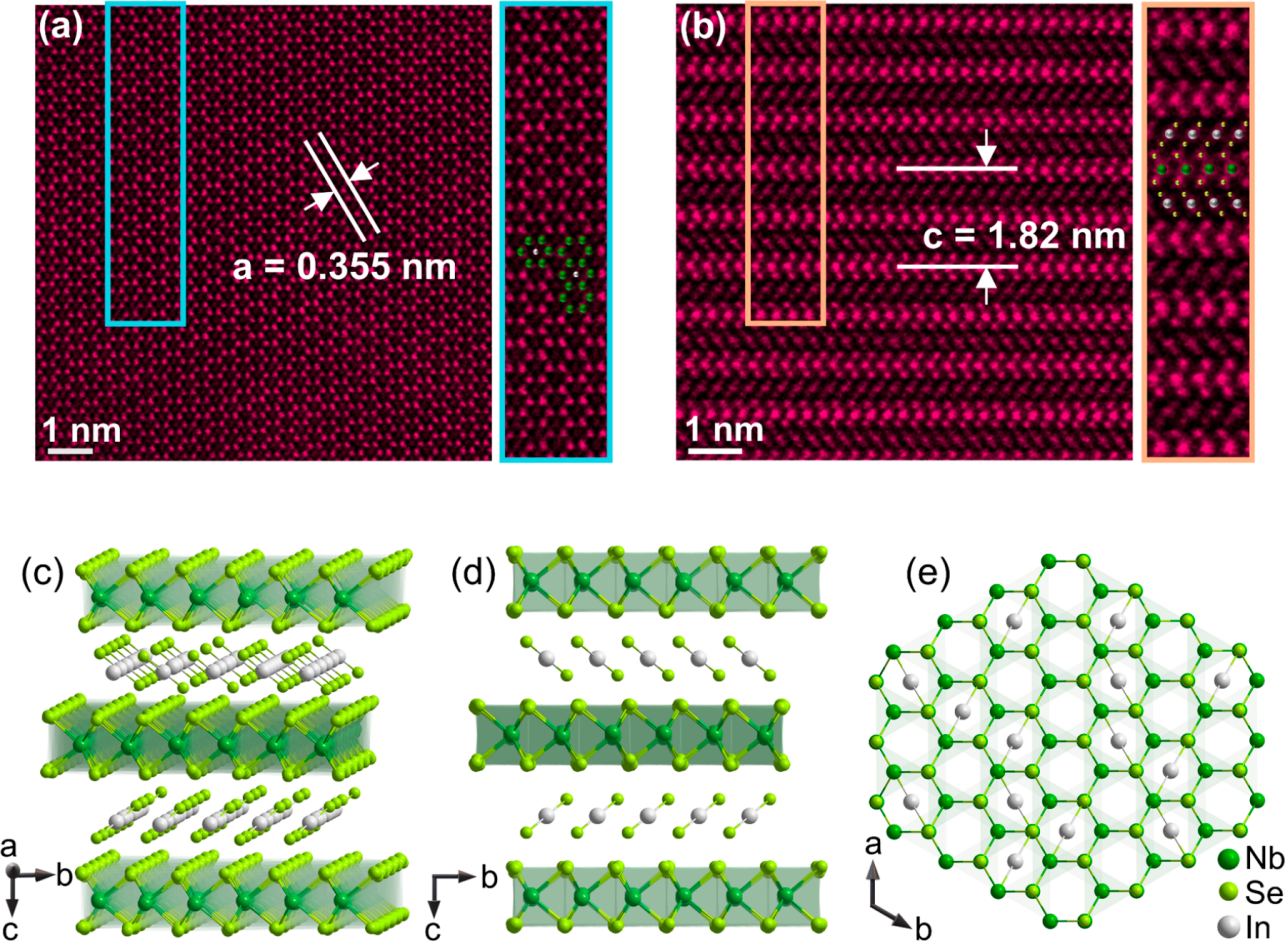
(a) Atomic-resolution transmission electron microscopy image of an (InSe_2_)_*x*_NbSe_2_ sample taken along the [001] zone-axis direction. (b) Atomic-resolution transmission electron microscopy image taken along the [100] zone-axis direction. (c) The crystal structure of InSe_2_-intercalated NbSe_2_. (d and e) The crystal structure of (InSe_2_)_*x*_NbSe_2_ within the *bc* plane and within the *ab* plane, respectively.

Figure 1b reveals that the obtained crystals are in a 2H phase with the In atoms intercalated in the van der Waals gaps of the NbSe_2_ lattice. Intriguingly, the In atoms do not exist as individual entities. Instead, they form bonds with two Se atoms along the *c*-axis of the crystals, creating bonded InSe_2_ intercalation layers. Due to the presence of intercalated InSe_2_ layers, the *c*-axis lattice constant experiences a significant elongation to 18.2 Å, approximately 50% larger than that of the pristine NbSe_2_ sample.^10^ This specific intercalation pattern results in the distinctive crystal structure observed in the (InSe_2_)_*x*_NbSe_2_ samples. In order to verify the change of lattice parameters, we performed X-ray diffraction (XRD) measurements on both the pristine NbSe_2_ and the intercalated samples. Figure S1 gives the single-crystal and powder XRD patterns of pristine and intercalated NbSe_2_. It is found that the *c*-axis lattice constant is elongated from 12.56 Å to 18.22 Å with the intercalation of an InSe_2_ layer, consistent with the HAADF-STEM results. A Rietveld refinement was performed on the powder XRD data of the NbSe_2_ and (InSe_2_)_*x*_NbSe_2_ samples. The typical refinement profiles and the resultant crystallographic information files are given in Figures S2–S4 and Tables S1 and S2, respectively.

From Figure 1b, it is evident that the brightness of the patterns of the InSe_2_ layers is less than that of the NbSe_2_ layers, indicating a discrepancy in site occupancy between the two. To determine the actual site occupancy, we conducted energy dispersive spectroscopy (EDS) measurements on the obtained crystals. Figure S5 presents the typical EDS analysis results for the samples grown at the NbSe_2_:In ratio of 1:2 to 1:7. The quantitative analysis of the EDS data reveals the chemical composition of (InSe_2_)_*x*_NbSe_2_, with the site occupancy rate (*x*) of the InSe_2_ intercalated layer ranging from 0.09 to 0.14 (Table S3). The observation of a low occupancy rate in the InSe_2_ intercalated layer is consistent with the HAADF-STEM results. It is also found that the Se site vacancy rates in the (InSe_2_)_*x*_NbSe_2_ samples (∼17%) are substantially larger than that of undoped TMD crystals (from Figure S6 it is seen that the Se site vacancy rate is ∼5% in an undoped NbSe_2_ single crystal).^24^ The enhanced Se site vacancy could originate from two facts: One is that some Se atoms escape from the NbSe_2_ layers to form an InSe_2_ bonded layer, resulting in more Se site vacancies compared to the pristine NbSe_2_ sample. The other one is that there are some individual In atoms that are inserted into the single-crystal samples.

Based on the HAADF-STEM and EDS results, we present the crystal structure of the (InSe_2_)_*x*_NbSe_2_ samples in Figure 1c. The intercalation of InSe_2_ layers leads to a significant elongation of the van der Waals gaps between two NbSe_2_ layers, resulting in a large *c*-axis lattice constant of 18.2 Å. In Figures 1d and 1e, we illustrate the crystal structures of (InSe_2_)_*x*_NbSe_2_ along the *bc* and *ab* planes, respectively. The intercalation of InSe_2_ bonded layers allows for maintenance of the sandwich-type stacking along the *c*-axis. An essential observation is the perfectly coincident zigzag orientations of the intercalated InSe_2_ bonded layers with the zigzag structures of the 2H phase NbSe_2_.

Figure 2a gives the temperature dependence of the resistivity measured within the basal *ab* plane of the (InSe_2_)_*x*_NbSe_2_ samples. At high temperature, the samples display metallic behavior. As the temperature decreases to around 11 K, superconductivity occurs. It is noted that the *T*_c_ value of the (InSe_2_)_*x*_NbSe_2_ samples is greatly enhanced compared to that of the pristine NbSe_2_ sample (see Figure S7; the *T*_c_ value of the NbSe_2_ sample is 6.7 K). Specifically, the *T*_c_ value is approximately 10.7 K for the (InSe_2_)_0.09_NbSe_2_ sample and approximately 10.2 K for (InSe_2_)_0.14_NbSe_2_. The highest transition temperature observed is approximately 11.6 K in the (InSe_2_)_0.12_NbSe_2_ sample. Hence, for the subsequent discussion in this work, we will focus on the properties of the (InSe_2_)_0.12_NbSe_2_ sample.

**Figure 2:**
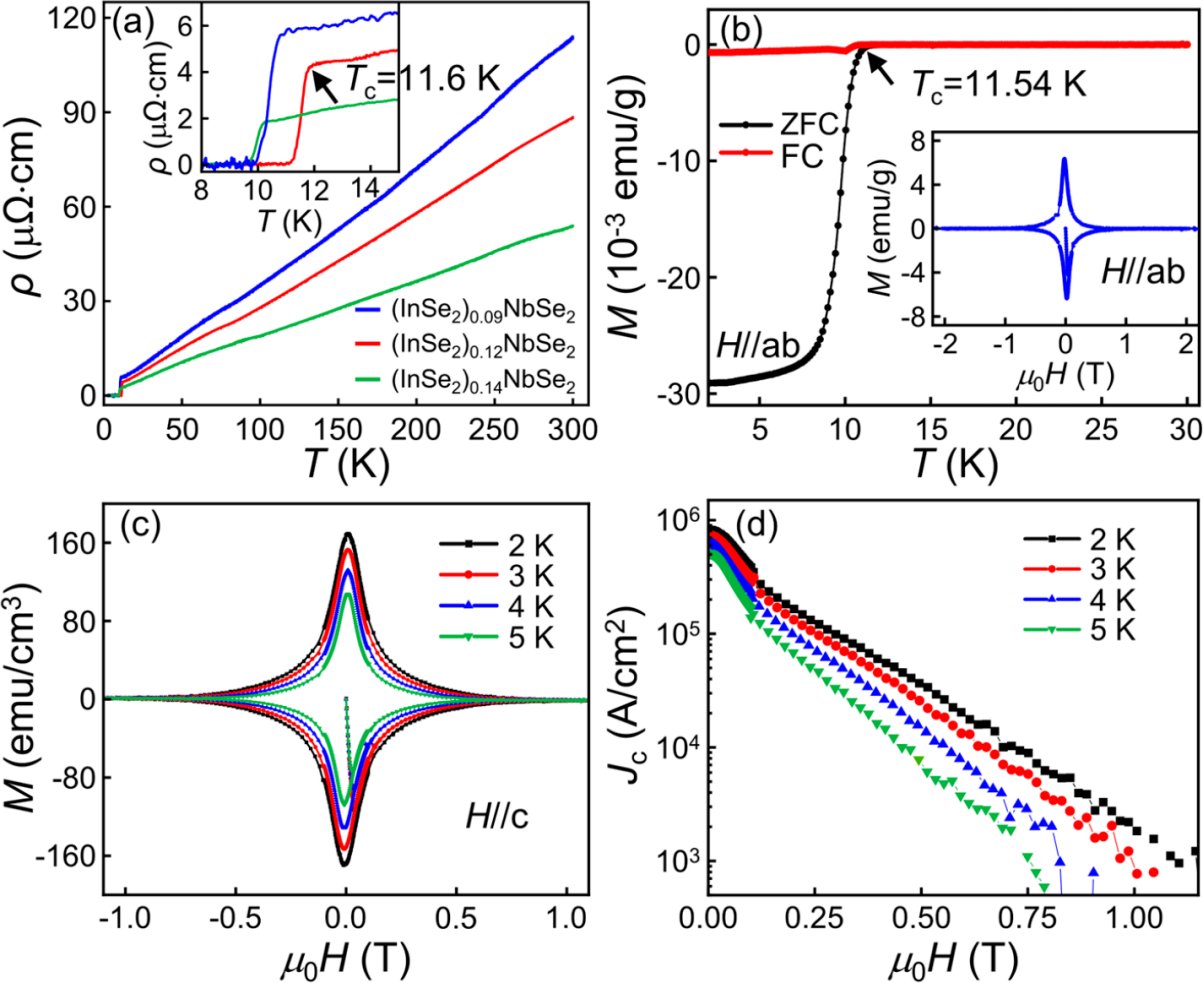
(a) The temperature dependence of in-plane resistivity of the (InSe_2_)_*x*_NbSe_2_ samples from *x* = 0.09 to *x* = 0.14. The inset shows an enlarged view of the superconducting transition. Here *T*_c_ is defined as the onset temperature where the sudden drop of resistivity occurs. (b) The temperature dependence of magnetic susceptibility of the (InSe_2_)_0.12_NbSe_2_ sample measured under both zero-field-cooling and field-cooling processes. The applied magnetic field is 2 Oe. Inset: The magnetic hysteresis loop at *T* = 2 K. (c) The magnetic field dependence of magnetization of the (InSe_2_)_0.12_NbSe_2_ sample at different temperatures. (d) The critical current density as a function of magnetic field for the (InSe_2_)_0.12_NbSe_2_ sample.

Figure 2b displays the temperature dependence of the magnetic susceptibility measured with the basal plane parallel to the magnetic field for the (InSe_2_)_0.12_NbSe_2_ sample. The *T*_c_ value determined from the magnetic data is 11.54 K. The estimated superconducting volume fraction is 94.2%, indicating that the (InSe_2_)_0.12_NbSe_2_ sample exhibits bulk superconductivity. Figure S8 shows the temperature dependence of the magnetic susceptibility for the (InSe_2_)_0.09_NbSe_2_ and (InSe_2_)_0.14_NbSe_2_ samples. The superconducting volume fractions for these samples are 86% and 75%, further confirming the occurrence of bulk superconductivity.

Figure S9 gives a comparison of the transition temperature of the (InSe_2_)_0.12_NbSe_2_ superconductor with those of previously reported TMD superconductors under different conditions. It is found that at ambient conditions the (InSe_2_)_0.12_NbSe_2_ sample exhibits the highest transition temperature among all TMD superconductors.

The critical current density (*J*_c_) is a crucial parameter that determines the application potential of a superconductor. To estimate the *J*_c_ value of the (InSe_2_)_0.12_NbSe_2_ sample, we use the Bean critical model,^25^
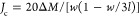
where Δ*M* is the width of the magnetization hysteresis loop and *w* and *l* represent the width and length of the sample, respectively. In Figure 2c, we plot the magnetization hysteresis loops of the (InSe_2_)_0.12_NbSe_2_ sample at different temperatures, with the applied magnetic field parallel to the *c*-axis of the sample. The resulting *J*_c_–μ_0_*H* curves are shown in Figure 2d. Notably, the *J*_c_ value reaches a remarkable value of 8.2 × 10^5^ A/cm^2^ at 2 K, which is four times larger than that observed in pure NbSe_2_.^26^ The *J*_c_ value of the (InSe_2_)_0.12_NbSe_2_ sample is larger than those of other TMD superconductors, such as NbS_2_, Cu_*x*_TiSe_2_, and Fe_*x*_NbSe_2_,^27−29^ and is comparable to other unconventional superconductors such as cuprate and iron-based superconductors.^30−33^

Figure 3a presents the magnetic field dependence of magnetization with *H*//*ab* from 2 to 12 K. In the Meissner shielding state (*H* < *H*_c__1_), the field dependence of the diamagnetic signal exhibits linear behavior. The corresponding lower critical field (*H*_c__1_) versus temperature data are summarized in Figure 3b. We use the phenomenological model *H*_c__1_(*T*) = *H*_c__1_ (0)[1 – (*T*/*T*_c_)^α^]^β^ to estimate the lower critical field. The resultant α and β are 1.68 and 2.5, and the *H*_c__1_(0) value is estimated to be 0.04 T. The deviation of the *H*_c__1_–*T* relation from the Ginzburg–Landau (G-L) scenario could be due to the two-dimensional character in (InSe_2_)_*x*_NbSe_2_ compounds.

**Figure 3:**
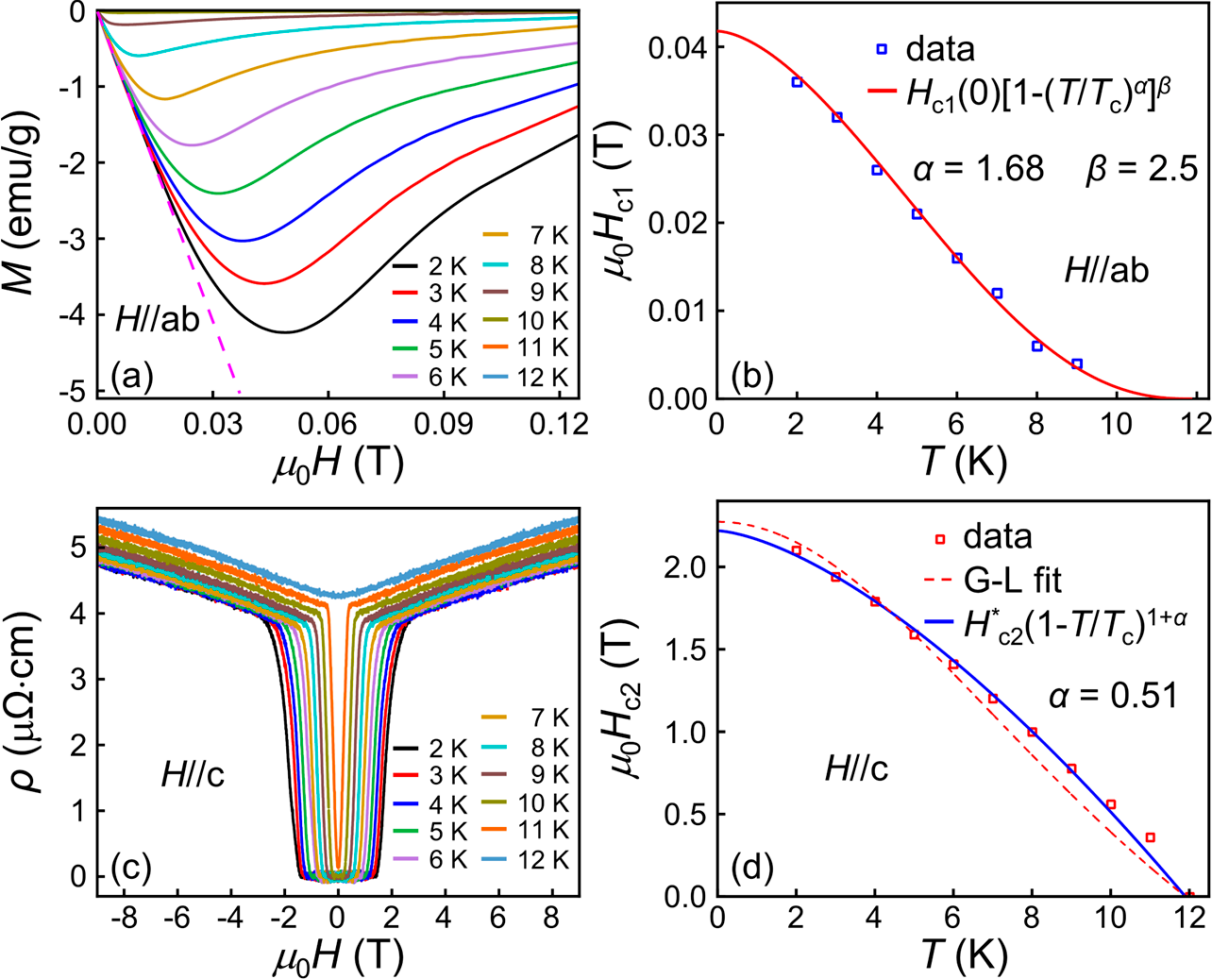
(a) The magnetic field dependence of magnetization at the low-field region of the (InSe_2_)_0.12_NbSe_2_ sample. (b) The lower critical field of the sample at different temperature. (c) The magnetic field dependence of resistivity at different temperatures near the superconducting transition. (d) The upper critical field of the (InSe_2_)_0.12_NbSe_2_ sample at different temperatures and the fitting according to the G-L formula and empirical formula.

Figure 3c shows the magnetic field dependence of the resistivity of the (InSe_2_)_0.12_NbSe_2_ sample at various temperatures near the superconducting transition. By employing the criterion of 90% of the normal-state resistance, we determined the upper critical field (*H*_c__2_). The temperature dependence of *H*_c__2_ is plotted in Figure 3d. We fit the *H*_c__2_–*T* relation according to the G-L formula *H*_c__2_(*T*) = *H*_c__2_(0)((1 – *t*^2^))/(1 + *t*^2^)) and the empirical formula *H*_c__2_(*T*) = *H*_c2_^*^(1 – *T*/*T*_c_)^1+α^, respectively. It is found that the empirical formula could well account for the upper critical field of the (InSe_2_)_0.12_NbSe_2_ sample. The *H*_c__2_–*T* relation of the (InSe_2_)_0.12_NbSe_2_ sample exhibits typical characteristic of clean-limit type-II superconductors, similar to that in Ba_3_NbS_5_-inserted 2H-NbS_2_.^34^ The coherence length of (InSe_2_)_0.12_NbSe_2_ is estimated to be ∼12.2 nm. And the magnetic penetration depth λ is estimated to be ∼104 nm.

To gain deeper insights into the enhancement of superconductivity and critical current density, we conduct first-principles calculations to examine the crystalline and electronic properties of (InSe_2_)_*x*_NbSe_2_.^35−40^Figure 4a illustrates the resulting crystalline structure obtained from self-consistent calculations. Notably, the *c*-axis lattice constant, 1.872 nm, closely matches the experimental value of 1.82 nm. Figures 4b and 4c show the top and side views of this crystalline structure, respectively. The sparse and disordered configuration of InSe_2_ with Se vacancies results in the light contrast of InSe_2_ layers, which is consistent with the HAADF-STEM results. To analyze the electronic properties, we simplify the crystalline structure by assuming an ordered stacking phase of (InSe_2_)_*x*_NbSe_2_ (details in Figures S10 and S11). In Figure 4d, we present the unfolding band structure, which closely resembles the case without intercalation (Figure S10b), with In atoms having no significant contribution to the states near the Fermi level (Figure 4e). Compared to the band structure of bulk 2H-NbSe_2_ (Figure S10c), the intercalation of the InSe_2_ layer enhances the two-dimensional features. The doping effect is relatively weak and cannot fully account for the observed enhancement of superconductivity. According to the BCS superconducting theory, we propose that the electron–phonon coupling is enhanced by the superstructure depicted in Figure 4a. We note that the rigidity of the superstructure is weakened by the presence of disordered stacking and partial InSe_2_ bonds. Consequently, some acoustic phonon modes can be softened, leading to an increased electron–phonon coupling and further enhancing superconductivity.

**Figure 4:**
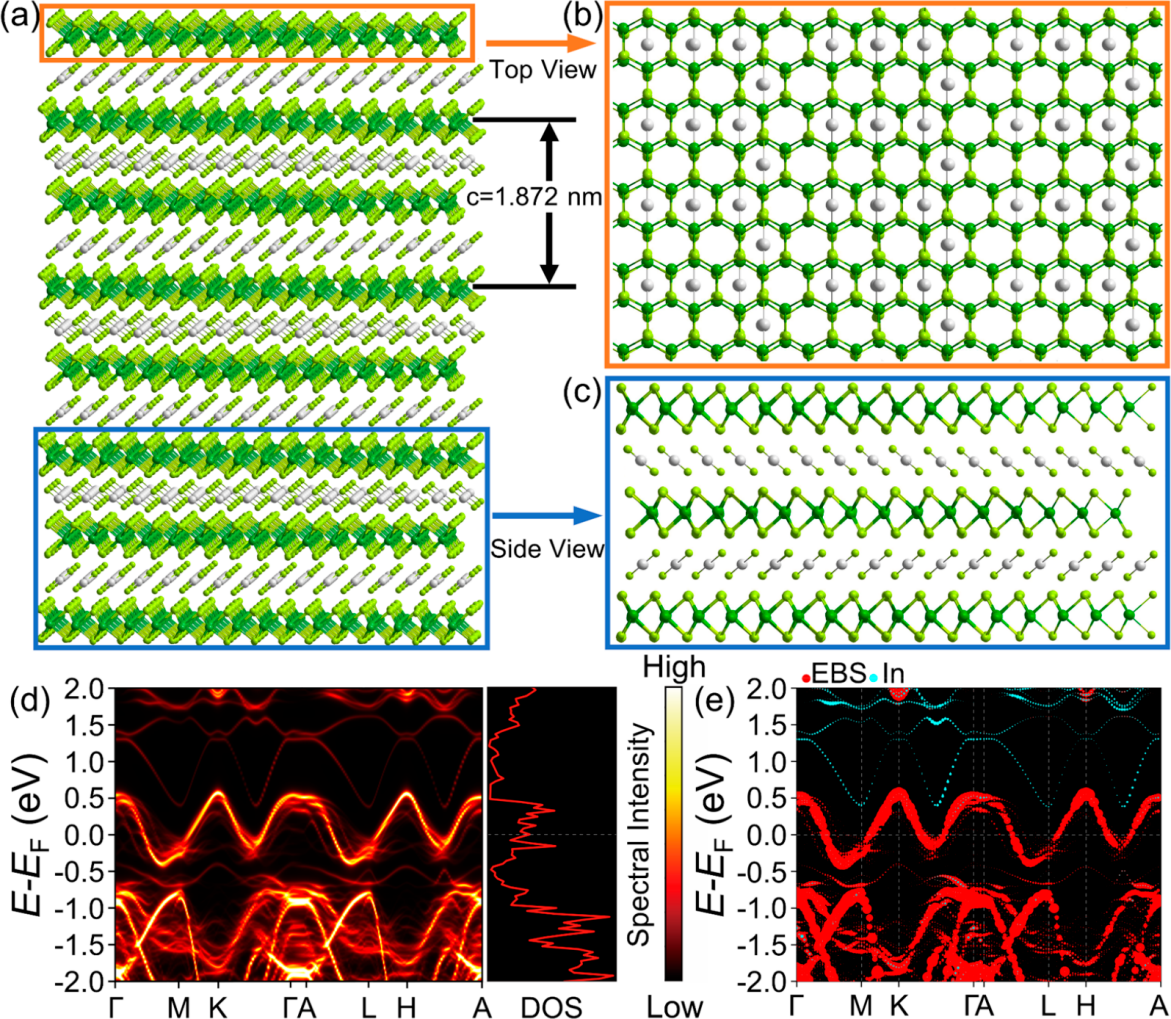
(a) The schematic diagram of (InSe_2_)_*x*_NbSe_2_. Due to the small ratio of InSe_2_ with a small amount of Se site vacancies, the InSe_2_ layers could be stacked in a random configuration. (b) The top view shows an irregular arrangement of In atoms in such a hexagonal lattice due to the randomly stacking InSe_2_ layers. (c) Two adjacent InSe_2_ layers align oppositely at the side view, which is consistent with the atomic arrangement observed in atomic-resolution HAADF-STEM in Figure 1b. (d) Unfolding effective band structure and density of states of an ordered stacking phase of (InSe_2_)_*x*_NbSe_2_ (see Figure S10 for details). (e) Projected effective band structure. The bands from the In atoms (cyan) hardly contribute to the Fermi surface.

In conclusion, we report the discovery of superconductivity in (InSe_2_)_*x*_NbSe_2_. Remarkably, the (InSe_2_)_0.12_NbSe_2_ superconductor displays enhanced superconductivity with a transition temperature of 11.6 K and critical current density of 8.2 × 10^5^ A/cm^2^. Our calculations reveal that the disordered stacking of InSe_2_ gives rise to a distinctive superstructure, providing an explanation for the observed enhancement of the superconducting performances. This finding opens an avenue for further exploration of new superconductors with superior performances in TMD materials.

## Supporting Information Available

Experimental details, supporting figures and tables, and computational details (PDF)
