# Supplementary material for: Enhanced Superconductivity and Critical Current Density Due to the Interaction of InSe2 Bonded Layer in (InSe2)0.12NbSe2
Source: J Am Chem Soc. 2024 Jan 5;146(2):1244–9. doi: 10.1021/jacs.3c09756 (PMC10797615; doi:10.1021/jacs.3c09756)
Supplement: Supplementary file 1 — ja3c09756_si_001.pdf [file ja3c09756_si_001.pdf]

Supporting Information for

Enhanced Superconductivity and Critical Current Density due to the Interaction of $InSe_2$ Bonded Layer in $(InSe_2)_{0.12}NbSe_2$

Rui Niu,[a,b,#] Jiayang Li,[a,b,#] Weili Zhen,[a,*] Feng Xu,[a] Shirui Weng,[a] Zhilai Yue,[a] Xiangmin Meng,[c] Jing Xia,[c,*] Ning Hao,[a,*] and Changjin Zhang[a,d,*]

[a]High Magnetic Field Laboratory of Anhui Province, HFIPS, Chinese Academy of Sciences, Hefei 230031, China

[b]Science Island Branch of Graduate School, University of Science and Technology of China, Hefei 230026, China

[c]Key Laboratory of Photochemical Conversion and Optoelectronic Materials, Technical Institute of Physics and Chemistry, Chinese Academy of Sciences, Beijing, 100190, China

[d]Collaborative Innovation Center of Advanced Microstructures, Nanjing University, Nanjing 210093, China

[#]R. Niu and J. Li contributed equally to this work.

[*]Emails: wlzhen@mail.ustc.edu.cn, xiajing@mail.ipc.ac.cn, haon@hmfl.ac.cn, zcjin@ustc.edu.cn

This file includes:

1. Experimental details.
2. Single crystal and powder X-ray diffraction patterns of the pristine and intercalated $NbSe_2$ samples.
3. Determining the chemical compositions of the samples.
4. Determining the Se site vacancy rate in pristine $NbSe_2$ single crystal.
5. Temperature dependence of resistivity of $NbSe_2$ single crystal
6. Magnetic susceptibility of the $(InSe_2)_{0.09}NbSe_2$ and $(InSe_2)_{0.14}NbSe_2$ samples showing the occurrence of bulk superconductivity.
7. A comparative diagram shown the superconducting transition temperature of TMD superconductors under different conditions.
8. Computational methods and calculation details.

1. Experimental details.

Growth of $NbSe_2$ and $(InSe_2)_xNbSe_2$ single crystals

The $(InSe_2)_xNbSe_2$ single crystals were grown using a two-step self-flux method. Firstly, we employed the chemical vapor transportation method with iodine as the transport agent to grow the single crystal of $NbSe_2$. Stoichiometric ratio of Nb powder, Se powder and 100 mg iodine were weight and sealed in an evacuated quartz tube. The quartz tube was placed in a two-zone furnace with the temperature setting at 800 ℃ and 725 ℃, respectively. The single crystal growth processes were maintained for two weeks. When the furnace is cooled down, shiny and black single crystals of $NbSe_2$ were obtained from the cold side of the tube. The dimensions of the pristine $NbSe_2$ single crystals could be as large as $0.4\times0.4\times0.05$ cm^3.

Subsequently, we placed the large pieces of $NbSe_2$ single crystal and indium pellets into an alumina crucible, which was then sealed under vacuum in a quartz tube. The molar ratio of $NbSe_2$:In was adjusted to range from 1:2 to 1:7. Here large amount of excess In was added because In plays dual roles of self-flux and insertions. The mixture of pellets was heated to 900 °C and maintained at that temperature for 3 days followed by slow cooling down to 600 °C. After that, the excess indium flux was removed through centrifuging, and the resultant samples were meticulously polished to eliminate any residual indium flux from the surfaces.

Characterization

For structural and compositional characterization, an atomic resolution microscope of JEOL ARM300F equipped with scanning transmission electron microscopy (STEM) and TEM ETA correctors was used. Single crystal and powder X-ray diffraction data were collected by a PANalytical X'Pert Pro MPD detector using monochromated Cu $K_{\alpha1}$ radiation as the X-ray source. Magnetization measurements were performed using a vibrating sample magnetometer (Quantum Design MPMS-3). Electrical measurements were conducted on a home-built Multiple Measurement System on a Janis-9T magnet. A four-point probe method was adopted in the electrical measurements. DC current of 1 mA was applied via a Keithley 6221 current source meter and the voltages were measured using a Keithley 2182A nanovoltmeter. Delta Mode measurements were used to improve signal-to-noise ratio.

2. Single crystal and powder X-ray diffraction patterns of the pristine and intercalated $NbSe_2$ samples.

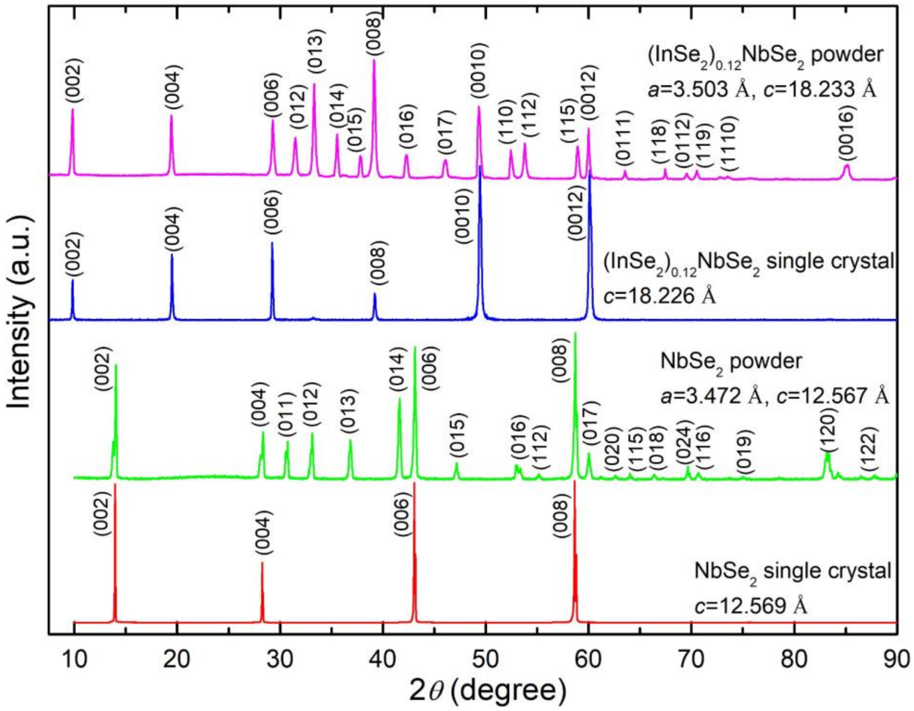

Figure S1. The single crystal and powder X-ray diffraction patterns of the pristine $NbSe_2$ and a typical $(InSe_2)_xNbSe_2$ sample. The wavelength of the irradiated X-ray is 1.5405 Å.

The *c*-axis lattice constant of the pristine $NbSe_2$ determined from the single crystal X-ray diffraction (XRD) pattern is 12.569 Å. And the lattice constants of $NbSe_2$ determined from the powder XRD pattern are a=3.472 Å and c=12.567 Å, respectively. These lattice parameters are consistent with those reported in literatures.

For the $(InSe_2)_xNbSe_2$ samples, the *a*-axis lattice constant is slightly larger than that of $NbSe_2$. Strikingly, the *c*-axis lattice constant is significantly enlarged with the intercalation of $InSe_2$. The *c*-axis lattice constant of the $(InSe_2)_{0.12}NbSe_2$ sample is about 18.22 Å, which is approximately 50% larger than that of undoped $NbSe_2$.

The lattice constants determined from XRD patterns are consistent with those calculated from the high-angle annular dark-field aberration-corrected scanning transmission electron microscopy (HAADF-STEM) images.

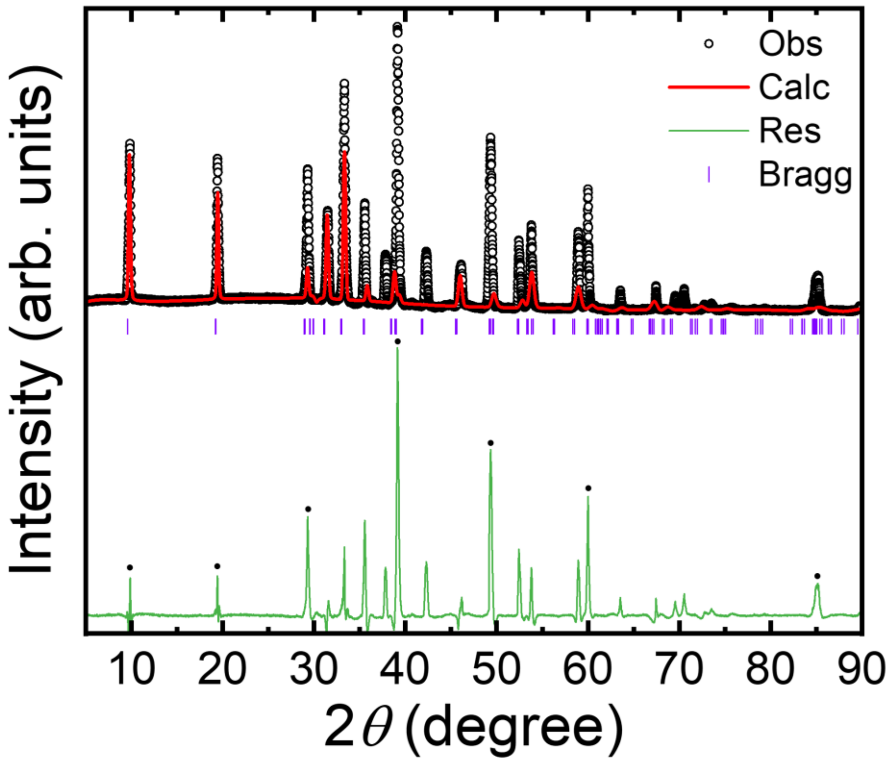

Figure S2. The Rietveld refinement results of the powder X-ray diffraction data of the $(InSe_2)_xNbSe_2$ sample. The black dots mark the positions of (00*l*) diffraction peaks, which reflect the preferred crystallographic orientation of the fine single crystal grains.

Table S1. Crystallographic data of $(InSe_2)_xNbSe_2$ based on the Rietveld refinements to the powder X-ray diffraction data. The large U_{iso} values could be due to the randomness of the intercalated $InSe_2$ bonds.

Formula	$(InSe_2)_{0.12}NbSe_2$
Space group	*P63/mmc*
a = *b* (Å)	3.501(4)
c (Å)	18.233(6)
α = *β* (°)	90
γ (°)	120

Atom	Multi.	*x*	*y*	*z*	Occ.	U_{iso}
Nb1	2	0.33333	0.66667	0.25	1.0	0.16449
Se1	4	0.66667	0.33333	0.15862	1.0	0.14664
Se2	4	0.33333	0.66667	0.44189	0.12	0.8
In	2	0	0	0.5	0.12	0.52247

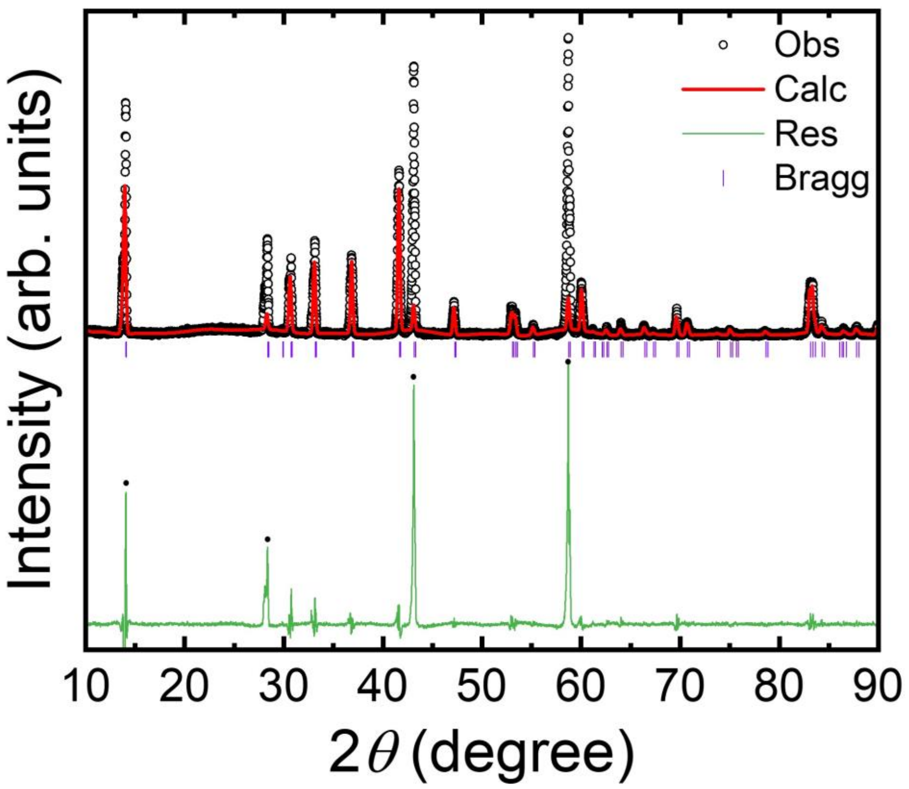

Figure S3. The Rietveld refinement results of the powder X-ray diffraction data of the pristine $NbSe_2$ sample. The black dots represent the diffraction peaks which are involving in the preferred crystallographic orientation.

Table S2. Crystallographic data of $NbSe_2$ based on the Rietveld refinements to the powder X-ray diffraction data.

Formula	$NbSe_2$
Space group	*P63/mmc*
$a = b$ (Å)	3.468(5)
c (Å)	12.562(7)
$\alpha = \beta$ (°)	90
γ (°)	120

Atom	Multi.	*x*	*y*	*z*	Occ.	U_{iso}
Nb1	2	0	0	0.25	1.0	0.00966
Se1	4	0.3333	0.6667	0.11882	1.0	0.00936

For some specific diffraction peaks, the difference between theoretical calculation and the experimental data is still a little bit large in the Rietveld refinement profile of the $(InSe_2)_{0.12}NbSe_2$ sample. This relatively large difference could come from two facts. The first one is that we are performing the powder X-ray diffraction measurement by grinding the pieces of single crystal samples into fine powder. Though we are trying our best to grind the single crystal samples, there could be some small single crystal grains which exhibiting some preferred crystallographic orientation. For examples, the large difference on the diffraction intensity of the patterns of (006), (008), (0010), (0012), and (0016) diffraction peaks could be due to the preferred crystallographic orientation. The second fact is that the intercalated $InSe_2$ could be randomly distributed in the van der Waals gaps. It is possible that there are some regions which have rich intercalated $InSe_2$ content and some regions with poor intercalated $InSe_2$ content, leading to more complicated crystallographic information. Further experimental measurements such as neutron diffraction and scanning tunnelling microscopy could contribute to a comprehensive understanding of the structural and physical properties of this and related materials.

For the pristine $NbSe_2$, the difference between theoretical calculation and the experimental data is also large for the (00*l*) diffraction peaks, which is due to the preferred orientation of the small single crystal grains.

Comparing the crystallographic data of the $(InSe_2)_{0.12}NbSe_2$ sample with those of the pristine $NbSe_2$ sample, it is found that the atomic positions of both Nb and Se1 exhibit some misfit. This misfit is reflected in the HAADF-STEM images and illustrated in Figure S4.

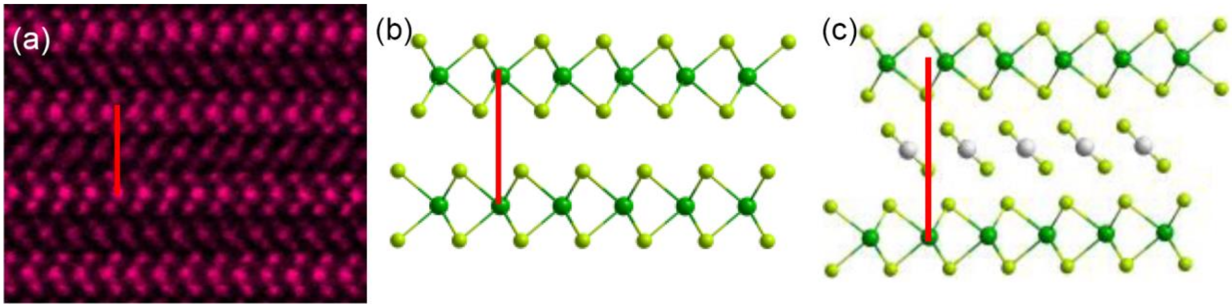

Figure S4. (a) A typical HAADF-STEM image of $(InSe_2)_xNbSe_2$ showing the misfit positions of Nb and Se in 2H-phase $NbSe_2$ due to the dragging of intercalated $InSe_2$ bonds. (b) The crystal structure of pristine $NbSe_2$. (c) The crystal structure of $(InSe_2)_xNbSe_2$.

3. Determining the chemical compositions of the samples.

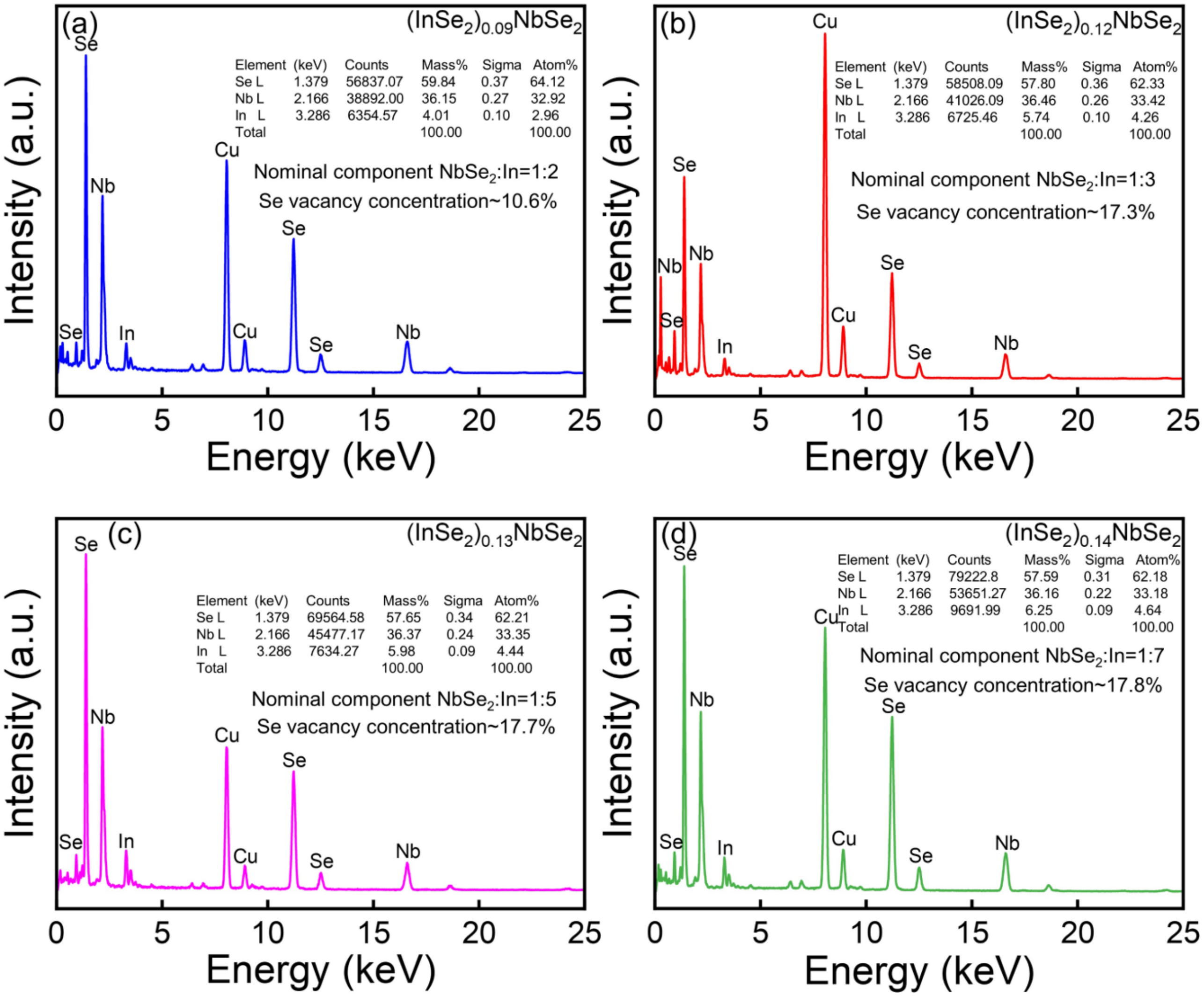

Figure S5. Typical energy dispersive X-ray spectroscopy data of the samples grown at $NbSe_2$:In ratio of (a) 1:2, (b) 1:3, (c) 1:5, and (d) 1:7. It is found that the real compositions of the samples could be written as (a) $(InSe_2)_{0.09}NbSe_2$, (b) $(InSe_2)_{0.12}NbSe_2$, (c) $(InSe_2)_{0.13}NbSe_2$ and (d) $(InSe_2)_{0.14}NbSe_2$. The presence of Cu is due to the utilization of Cu mesh during the measurements. And Cu is removed from the chemical composition analyses.

It is noted that there is a substantial amount of Se site vacancy (~17%) in the $(InSe_2)_xNbSe_2$ samples. In order to learn more about the Se site vacancy, we perform the energy dispersive X-ray spectroscopy (EDS) experiments on the pristine $NbSe_2$ single crystal (Figure S6). The Se-site vacancy of the $NbSe_2$ sample is determined to be about 5%. The presence of more Se-site vacancy in $(InSe_2)_xNbSe_2$ could be explained according to the following two possibilities: The first one is that we are growing the $(InSe_2)_xNbSe_2$ single crystals using the $NbSe_2$ single crystal and In as the starting materials. Some Se atoms are probably escaped from the $NbSe_2$ layers to form the $InSe_2$ layers, leaving more Se site vacancy in the $NbSe_2$ layers. The second one is that there might be some isolated In atoms which are inserted into the Van der Waals gaps of 2H-phase $NbSe_2$. The presence of isolated In atoms could be further checked by a comprehensive scanning tunneling microscopy measurement.

Table S3. The quantitative EDS analyses of the chemical compositions of $(InSe_2)_xNbSe_2$ samples grown with the $NbSe_2$:In molar ratio between 1:2 and 1:7.

Initial ratio between $NbSe_2$ and In	Se atom (%)	Nb atom (%)	In atom (%)	Chemical composition
1:2	64.12	32.92	2.96	$(InSe_2)_{0.09}NbSe_2$
1:2.5	63.05	33.17	3.78	$(InSe_2)_{0.11}NbSe_2$
1:3	62.33	33.42	4.26	$(InSe_2)_{0.12}NbSe_2$
1:4	62.25	33.41	4.34	$(InSe_2)_{0.13}NbSe_2$
1:5	62.21	33.35	4.44	$(InSe_2)_{0.13}NbSe_2$
1:6	62.15	33.29	4.56	$(InSe_2)_{0.14}NbSe_2$
1:7	62.18	33.18	4.64	$(InSe_2)_{0.14}NbSe_2$

4. Determining the Se site vacancy rate in pristine $NbSe_2$ single crystal

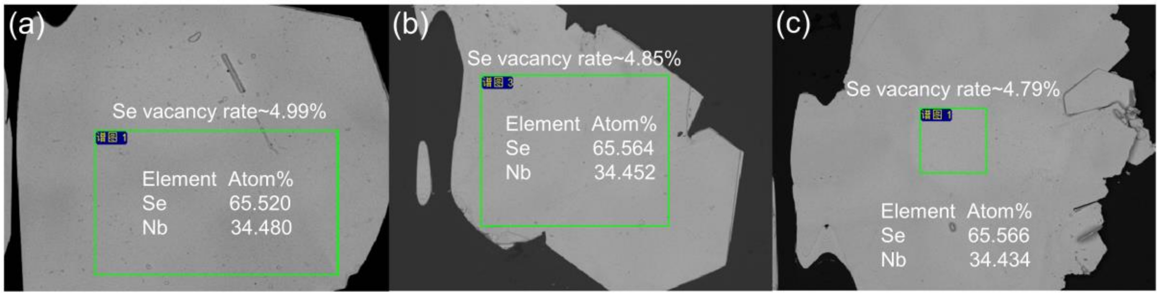

Figure S6. The Scanning electron microscope patterns of three pieces of $NbSe_2$ single crystals. The quantitative analyses of the EDS data are shown in the images. The Se site vacancy rate is approximately 5% in pristine $NbSe_2$ single crystals.

5. Temperature dependence of resistivity of $NbSe_2$ single crystal.

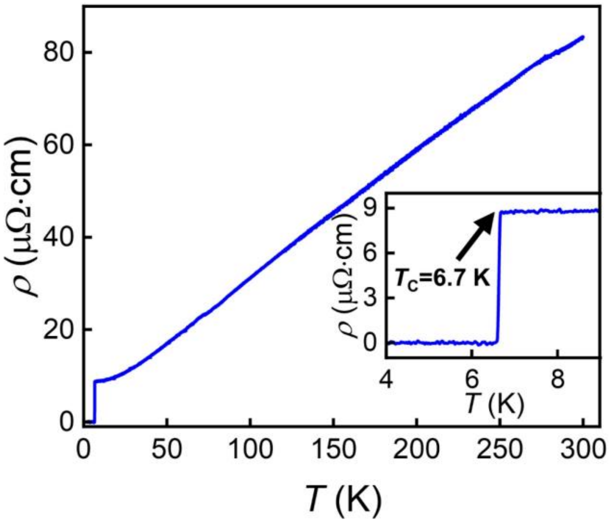

Figure S7. Temperature dependence of in-plane resistivity of $NbSe_2$ sample.

6. Magnetic susceptibility of the $(InSe_2)_{0.09}NbSe_2$ and $(InSe_2)_{0.14}NbSe_2$ samples showing the occurrence of bulk superconductivity.

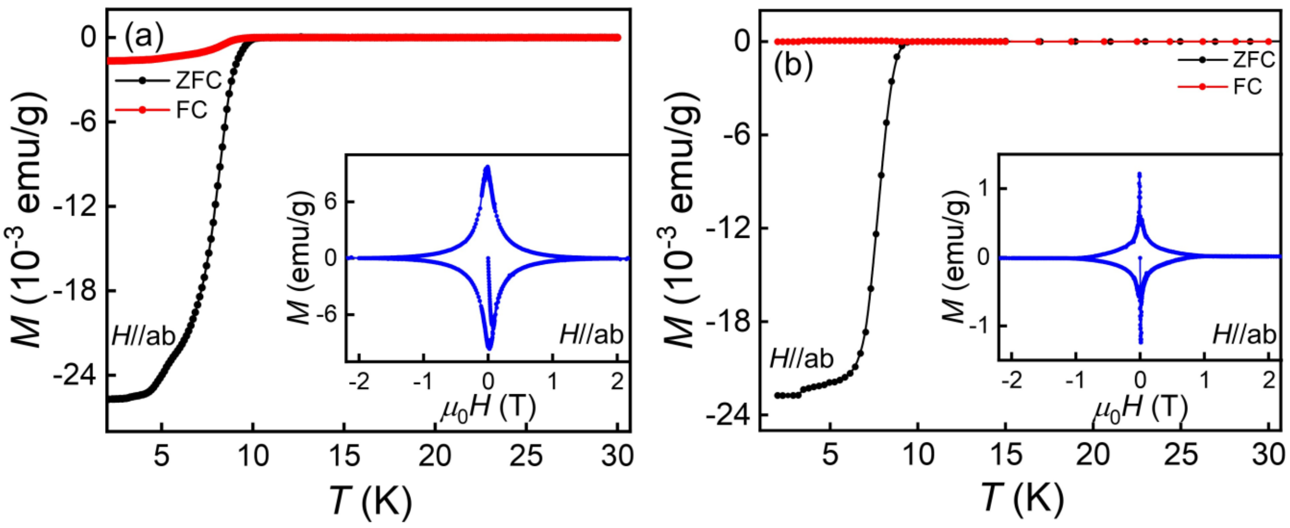

Figure S8. Temperature dependence of magnetic susceptibility for the (a) $(InSe_2)_{0.09}NbSe_2$ and (b) $(InSe_2)_{0.14}NbSe_2$ samples. The magnetic field of 2 Oe is applied parallel to the *ab* plane. Insets: The magnetic hysteresis loop at T=2 K.

7. A comparative diagram shown the superconducting transition temperature of TMD superconductors under different conditions.

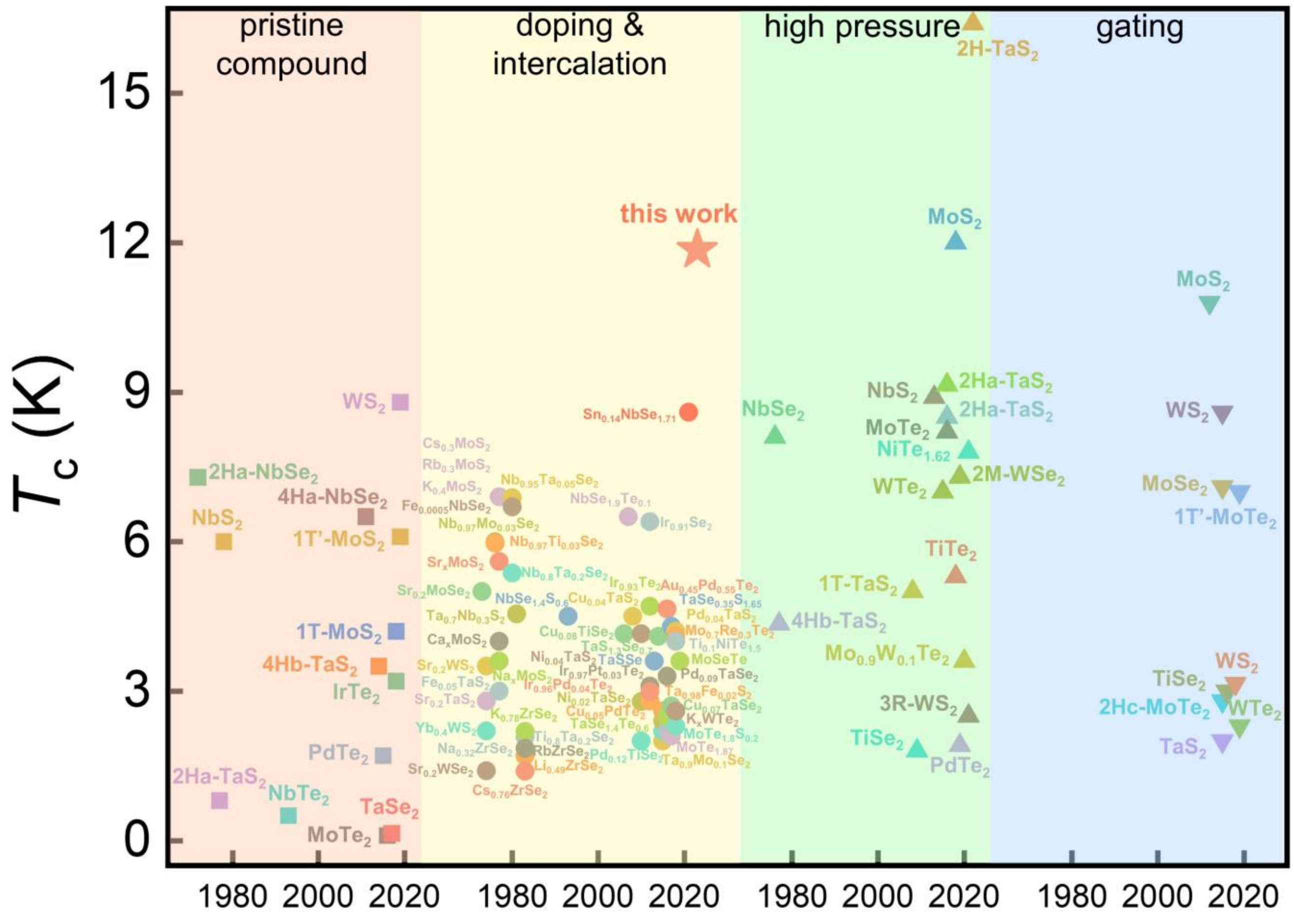

Figure S9. A comparison of the superconducting transition temperature between this work and previous reports.[1-37] The superconducting transition temperature of $(InSe_2)_{0.12}NbSe_2$ is the highest among all TMD superconductors under ambient pressure. It is noted that the transition temperature of 2H-TaS_2 under high pressure is higher than the present work.[21]

8. Computational methods and calculation details.

The calculations of geometrical relaxations and electronic properties were based on density functional theory (DFT), performed by using the Vienna *Ab-initio* Simulation Package (VASP).[38] We used the projector augmented wave (PAW) pseudopotentials with the Perdew-Burke-Ernzerhof (PBE) exchange-correlation functional in the generalized gradient approximation.[39,40] To correct the dispersive interactions in the van der Waals materials, the semi-empirical DFT-D3 method were employed under the whole process of calculations.[41] The kinetic energy cutoff of the plane-wave basis was chosen to be 500 eV. The self-consistent energy was converged within 10^{-6} eV, and optimized atomic structures were achieved when forces on all the atoms were smaller than 5×10^{-3} eV/Å. A Γ-centered 18×18×18 Monkhorst-Pack (MP) k-mesh was used to sample the Brillouin zone for density of states (DOS) calculations of $2H_a$-$NbSe_2$ and 10×10×10 MP k-mesh for DOS calculations of supercell after In intercalation.[42] The band unfolding program KPROJ was used to process the band structures of supercell.[43]

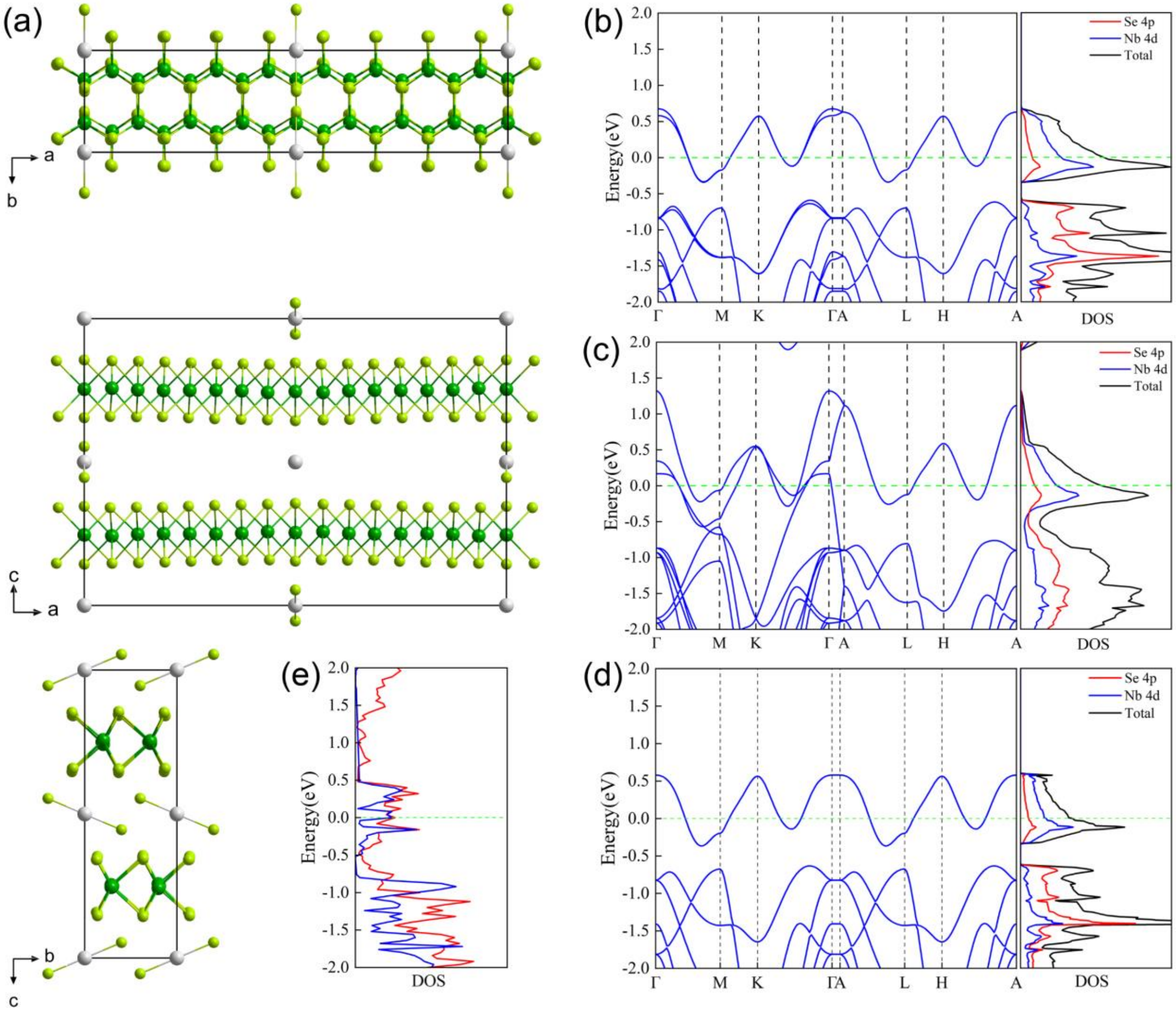

Figure S10. (a) Crystal structure of an ordered phase of $(InSe_2)_xNbSe_2$ with Se:Nb:In=68:32:4. The *c*-axis lattice constant is determined to be 18.72 Å after geometric optimization, which is consistent with the experimental data. (b) The band structure and density of states for primitive cell of $NbSe_2$ without intercalation. (c) The band structure and density of states for bulk $2H_a$-$NbSe_2$. (d) The band structure and density of states for monolayer $2H_a$-$NbSe_2$. (e) Comparison of density of states with/without $InSe_2$ layers based on crystal structure (a). The density of states near Fermi level does not change significantly with (red line) of without (bule line) $InSe_2$ layers.

Figure S10 is obtained from the self-consistent calculations according to the experimentally determined lattice parameters. Note that some individual In atoms should exist to maintain the atom ratio of Se:Nb:In=68:32:4 and to have the *c*-axis lattice constant be close to the experimentally determined value (~1.826 nm). The theoretically calculated data support that there are some individual In atoms which are inserted in the $NbSe_2$ compound, which are consistent with the energy dispersive X-ray spectroscopy results.

Other similar structures without individual In atoms could lead to either a *c*-axis of more than 2 nm (about 10% relative error) or deformed lattice structures after geometric relaxation. As shown in Figure S10, the *c*-axis lattice parameter could be 2.03 nm if we do not consider any individual In atoms, which is much larger than the experimental value. If a uniaxial pressure of 3 GPa is artificial applied along the *c*-axis, a metastable structure with smaller *c*-axis lattice constant of 1.81 nm could be obtained.

The electronic structure shown in Figure S11 is nearly unchanged in comparison with the case in Figure 4. Thus, we suggest that the metastable structure shown in Figure S10c is a likely structure. However, the structure shown in Figure 4 and Figure S10 is the most probable one, which naturally follows all the experimental requirements without any artificial modulations.

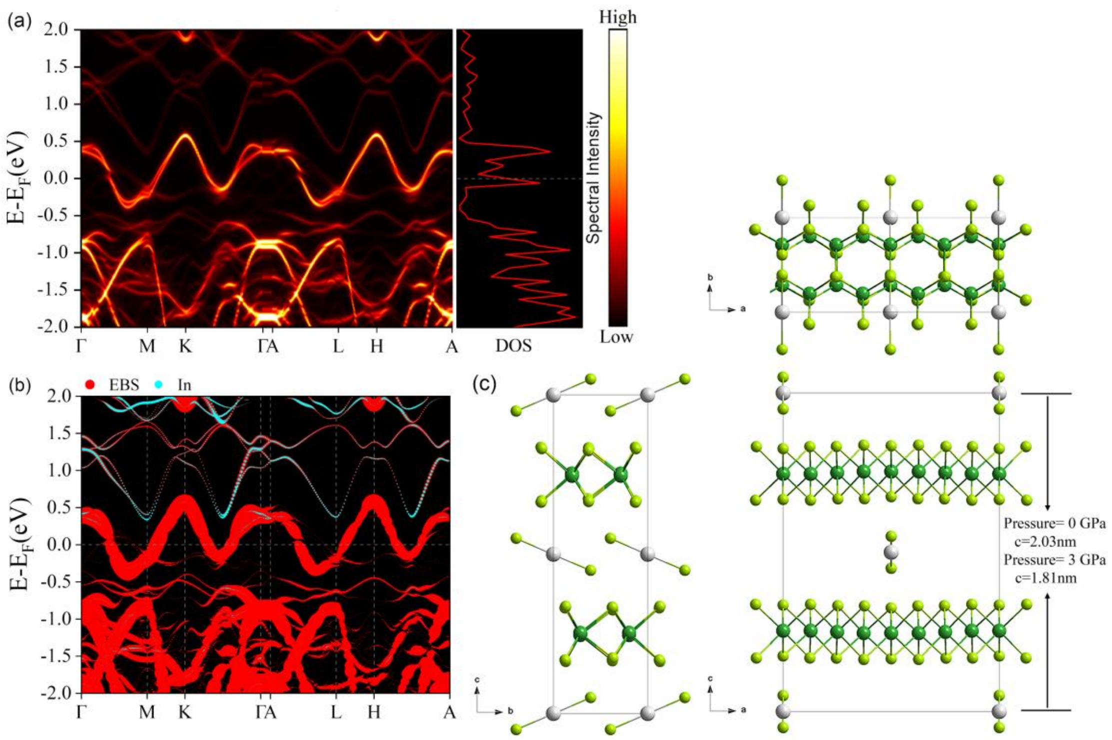

Figure S11. (a) Unfolding effective band structure (EBS) and density of states (DOS) of an ordered stacking phase of $(InSe_2)_xNbSe_2$ shown in (c). (b) Projected effective band structure. The bands coming from In atoms (cyan) have no contributions to the Fermi surface. (c) A structure of $(InSe_2)_xNbSe_2$ without individual In atoms.

References:

(1) Pan, J.; Guo, C.; Song, C.; et al. Enhanced superconductivity in restacked TaS_2 nanosheets. *J. Am. Chem. Soc.* **2017**, 139, 4623−4626.

(2) Fang, Y.; Pan, J.; Zhang, D; et al. Discovery of superconductivity in 2M WS_2 with possible topological surface states. *Adv. Mater.* **2019**, 31, 1901942.

(3) Morris, R. C.; Coleman, R. V. Superconductivity and magnetoresistance in $NbSe_2$. *Phys. Rev. B* **1972**, 5, 895-901.

(4) Onabe, K.; Naito, M; Tanaka, S. Anisotropy of upper critical field in superconducting 2H-NbS_2. *J. Phys. Soc. Jpn.* **1978**, 45, 50-58.

(5) Peng, J.; Liu, Y; Luo, X; et al. High phase purity of large-sized 1T'-MoS_2 monolayers with 2D superconductivity. *Adv. Mater.* **2019**, 31, 1900568.

(6) Morosan, E.; Zandbergen, H. W.; Dennis, B. S.; et al. Superconductivity in Cu_xTiSe_2. *Nat. Phys.* **2006**, 2, 544-550.

(7) Yamamoto, M.; Sambongi, T. Superconducting transition temperature and magnetic susceptibility in the solid solutions $Nb_{1-x}Mo_xSe_2$ and $Nb_{1-y}Ti_ySe_2$. *J. Phys. Soc. Jpn.* **1976**, 41, 1146-1152.

(8) Zhou, M.; Li, X; Dong, C. Superconductivity in palladium-doped 2H-TaS_2. *Supercond. Sci. Technol.* **2018**, 31, 065001.

(9) Woollam, J. A.; Somoano, R. B. Physics and chemistry of MoS_2 intercalation compounds. *Mater. Sci. Eng.* **1977**, 31, 289-295.

(10) Subba Rao, G. V.; Shafer, M. W.; Kawarazaki, S.; et al. Superconductivity in alkaline earth metal and Yb intercalated group VI layered dichalcogenides. *J. Solid State Chem.* **1974**, 9, 323-329.

(11) Kudo, K.; Ishii, H; Nohara, M. Composition-induced structural instability and strong-coupling superconductivity in $Au_{1-x}Pd_xTe_2$. *Phys. Rev. B* **2016**, 93, 140505(R).

(12) Qi, Y.; Matsuishi, S.; Guo, J.; et al. Superconductivity in defective pyrite-type iridium chalcogenides Ir_xCh_2 (Ch=Se and Te). *Phys. Rev. Lett.* **2012**, 109, 217002.

(13) Pyon, S.; Kudo, K.; Nohara, M. Superconductivity induced by bond breaking in the triangular lattice of $IrTe_2$. *J. Phys. Soc. Jpn.* **2012**, 81, 053701.

(14) Mandal, M.; Marik, S.; Sajilesh, K. P.; et al. Enhancement of the superconducting transition temperature by Re doping in Weyl semimetal $MoTe_2$. *Phys. Rev. Mater*. **2018**, 2, 094201.

(15) Chi, Z.; Chen, X.; Yen, F.; et al. Superconductivity in pristine $2H_a$-MoS_2 at ultrahigh pressure. *Phys. Rev. Lett.* **2018**, 120, 037002.

(16) Morosan, E.; Wagner, K. E.; Zhao, L.; et al. Multiple electronic transitions and superconductivity in Pd_xTiSe_2. *Phys. Rev. B* **2010**, 81, 094524.

(17) Luo, H.; Xie, W.; Tao, J.; et al. Differences in chemical doping matter: superconductivity in $Ti_{1-x}Ta_xSe_2$ but not in $Ti_{1-x}Nb_xSe_2$. *Chem. Mater.* **2016**, 28, 1927-1935.

(18) Wagner, K. E.; Morosan, E.; Hor, Y. S.; et al. Tuning the charge density wave and superconductivity in Cu_xTaS_2. *Phys. Rev. B* **2008**, 78, 104520.

(19) Zhang, W.; Fang, Y.; Zhang, Z.; et al. A new superconducting 3R-WS_2 phase at high pressure. *J. Phys. Chem. Lett.* **2021**, 12, 3321-3327.

(20) Fang, Y.; Dong, Q.; Pan, J.; et al. Observation of superconductivity in pressurized 2M WSe_2 crystals. *J. Mater. Chem. C* **2019**, 7, 8551.

(21) Dong, Q.; Pan, J.; Li, S.; et al. Record-High superconductivity in transition metal dichalcogenides emerged in compressed 2H-TaS_2. *Adv. Mater.* **2022**, 34, 2103168.

(22) Kusmartseva, A. F.; Sipos, B.; Berger, H.; et al. Pressure induced superconductivity in pristine 1T-$TiSe_2$. *Phys. Rev. Lett.* **2009**, 103, 236401.

(23) Deng, Y.; Lai, Y.; Zhao, X.; et al. Controlled growth of 3R phase tantalum diselenide and its enhanced superconductivity. *J. Am. Chem. Soc*. **2020**, 142, 2948−2955.

(24) Sipos, B.; Kusmartseva, A. F.; Akrap, A.; et al. From Mott state to superconductivity in 1T-TaS_2. *Nat. Mater.* **2008**, 7, 960-965.

(25) Freitas, D. C.; Rodière, P.; Osorio, M. R.; et al. Strong enhancement of superconductivity at high pressures within the charge-density-wave states of 2H-TaS_2 and 2H-$TaSe_2$. *Phys. Rev. B* **2016**, 93, 184512.

(26) Berthier, C.; Molinié, P.; Jérome, D. Evidence for a connection between charge density waves and the pressure enhancement of superconductivity in 2H-$NbSe_2$. *Solid State Commun*. **1976**, 18, 1393-1395.

(27) Tissen, V. G.; Osorio, M. R.; Brison, J. P.; et al. Pressure dependence of superconducting critical temperature and upper critical field of 2H-NbS_2. *Phys. Rev. B* **2013**, 87, 134502.

(28) Qi, Y.; Naumov, P. G.; Ali, M. N.; et al. Superconductivity in Weyl semimetal candidate $MoTe_2$. *Nat. Commun.* **2016**, 7, 11038.

(29) Pan, X.; Chen, X.; Liu, H.; et al. Pressure-driven dome-shaped superconductivity and electronic structural evolution in tungsten ditelluride. *Nat. Commun.* **2015**, 6, 7805.

(30) Dong, Q.; Pan, J.; Li, S.; et al. Abnormal metal-semiconductor-like transition and exceptional enhanced superconducting state in pressurized restacked TaS_2. *J. Am. Chem. Soc*. **2023**, 145, 14581-14586.

(31) Dahal, R.; Deng, L.; Poudel, N.; et al. Tunable structural phase transition and superconductivity in the Weyl semimetal $Mo_{1-x}W_xTe_2$. *Phys. Rev. B* **2020**, 101, 140505(R).

(32) Feng, Z.; Si, J.; Li, T.; et al. Evidences for pressure-induced two-phase superconductivity and mixed structures of $NiTe_2$ and NiTe in type-II Dirac semimetal $NiTe_{2-x}$ (x=0.38 ± 0.09) single crystals. *Mater. Today Phys.* **2021**, 17, 100339.

(33) Gu, K.; Susilo, R. A.; Ke, F.; et al. Pressure-induced enhancement in the superconductivity of $ZrTe_3$. *J. Phys. Condens. Matter* 2018, 30, 385701.

(34) Yu, Y.; Yang, F.; Lu, X.; et al. Gate-tunable phase transitions in thin flakes of 1T-TaS_2. *Nat. Nanotechnol.* 2015, 10, 270-276.

(35) Ye, J.; Zhang, Y.; Akashi, R.; et al. Superconducting dome in a gate-tuned band insulator. *Science* 2012, 30, 1193-1196.

(36) Lu, J.; Zheliuk, O.; Chen, Q.; et al. Full superconducting dome of strong Ising protection in gated monolayer WS_2. *Proc. Natl. Acad. Sci. U.S.A.* 2018, 115, 3551-3556.

(37) Zhang, H.; Rousuli, A.; Shen, S.; et al. Enhancement of superconductivity in organic-inorganic hybrid topological materials. *Sci. Bull.* 2020, 65, 188-193.

(38) Kresse, G.; Furthmüller, J. Efficient iterative schemes for ab initio total-energy calculations using a plane-wave basis set. *Phys. Rev. B* **1996**, 54, 11169-11186.

(39) Blöchl, P. E. Projector augmented-wave method. *Phys. Rev. B* **1994**, 50, 17953-17979.

(40) Perdew, J. P.; Burke, K.; Ernzerhof, M. Generalized gradient approximation made simple. *Phys. Rev. Lett.* **1996**, 77, 3865-3868.

(41) Grimme, S.; Antony, J.; Ehrlich, S.; Krieg, H. A consistent and accurate ab initio parametrization of density functional dispersion correction (DFT-D) for the 94 elements H-Pu. *J. Chem. Phys.* **2010**, 132, 154104.

(42) Methfessel M.; Paxton, A. T. High-precision sampling for Brillouin-zone integration in metals. *Phys. Rev. B* **1989**, 40, 3616-3621.

(43) Chen, M.; Weinert, M. Layer k-projection and unfolding electronic bands at interfaces. *Phys. Rev. B* **2018**, 98, 245421.
